# A positive feedback loop between gastric cancer cells and tumor-associated macrophage induces malignancy progression

**DOI:** 10.1186/s13046-022-02366-6

**Published:** 2022-05-14

**Authors:** Haiyan Piao, Lingfeng Fu, Yuxin Wang, Yang Liu, Yue Wang, Xiangyu Meng, Dong Yang, Xiang Xiao, Jun Zhang

**Affiliations:** 1 Medical Oncology Department of Gastrointestinal Cancer, Liaoning Province Cancer Hospital & Institute (Cancer Hospital of China Medical University), No. 44 Xiaoheyan Road, Dadong District, Shenyang, 110042 Liaoning China; 2 Department of Gastroenterological Surgery, Graduate School of Life Sciences, Kumamoto University, 1-1-1 Honjo, Chuo-ku, Kumamoto, 860-8556 Japan; 3 Gastrointestinal Cancer Biology, International Research Center for Medical Sciences, Kumamoto University, Kumamoto, Japan; 4 Department of Hematology, Zhujiang Hospital, Southern Medical University, Guangzhou, China; 5 Laboratory of Stem Cell Stress, International Research Center for Medical Sciences (IRCMS), Kumamoto University, Kumamoto, Japan; 6 Department of Oncology, Shengjing Hospital of China Medical University, No. 36 Sanhao Street, Heping District, Shenyang, 110004 Liaoning China; 7 Gastric Cancer Department, Liaoning Province Cancer Hospital & Institute (Cancer Hospital of China Medical University), No. 44 Xiaoheyan Road, Dadong District, Shenyang, 110042 Liaoning China; 8 Shanghai Yanji Biomedical Technology, Shanghai, China

**Keywords:** Gastric cancer, Macrophage, Positive feedback loop, Hypoxia, Cytokines, Signaling pathway

## Abstract

**Background:**

Hypoxia and inflammation tumor microenvironment (TME) play a crucial role in tumor development and progression. Although increased understanding of TME contributed to gastric cancer (GC) progression and prognosis, the direct interaction between macrophage and GC cells was not fully understood.

**Methods:**

Hypoxia and normoxia macrophage microarrays of GEO database was analyzed. The peripheral blood mononuclear cell acquired from the healthy volunteers. The expression of C-X-C Motif Chemokine Ligand 8 (*CXCL8*) in GC tissues and cell lines was detected by quantitative reverse transcription PCR (qRT-PCR), western-blot, Elisa and immunofluorescence. Cell proliferation, migration, and invasion were evaluated by cell counting kit 8 (CCK8), colony formation, real-time imaging of cell migration and transwell. Flow Cytometers was applied to identify the source of cytokines. Luciferase reporter assays and chromatin immunoprecipitation were used to identify the interaction between transcription factor and target gene. Especially, a series of truncated and mutation reporter genes were applied to identify precise binding sites. The corresponding functions were verified in the complementation test and in vivo animal experiment.

**Results:**

Our results revealed that hypoxia triggered macrophage secreted *CXCL8*, which induced GC invasion and proliferation. This macrophage-induced GC progression was *CXCL8* activated C-X-C Motif Chemokine Receptor 1/2 (*CXCR1/2*) on the GC cell membrane subsequently hyperactivated Janus kinase 1/ Signal transducer and activator of transcription 1 (*JAK/STAT1*) signaling pathway. Then, the transcription factor *STAT1* directly led to the overexpression and secretion of Interleukin 10 (*IL-10*). Correspondingly, *IL-10* induced the M2-type polarization of macrophages and continued to increase the expression and secretion of *CXCL8*. It suggested a positive feedback loop between macrophage and GC. In clinical GC samples, increased *CXCL8* predicted a patient’s pessimistic outcome.

**Conclusion:**

Our work identified a positive feedback loop governing cancer cells and macrophage in GC that contributed to tumor progression and patient outcome.

**Supplementary Information:**

The online version contains supplementary material available at 10.1186/s13046-022-02366-6.

## Background

Gastric cancer (GC) is a prevalent yet incurable malignancy of the digestive system. It is the 3rd leading cause of cancer-related mortalities across the globe [1]. China has a high GC incidence and a heavy disease burden, with an estimated 320,000 annual deaths, accounting for 45% of all GC deaths globally [2]. Despite the constant iterations of comprehensive therapy based on surgical resection, GC prognosis remains poor, with a 5-year survival rate of not more than 30% [3]. On one hand, this is attributed to the malignant phenotype of the tumor; while on the other hand, the tumor microenvironment plays an essential role as an “accomplice” [4]. Hypoxia and inflammation are essential components of the tumor microenvironment (TME) [5, 6]. Our previous studies confirmed that hypoxia promotes GC progression [7]. Moreover, the hypoxia-related core element, hypoxia-inducible factor-1α (*HIF-1α*) were confirmed as transcription factors that could broadly regulate the transcription of downstream genes, promoting GC proliferation [8] and invasion [9, 10]. Therefore, it is essential to further explore the crosstalk between hypoxia and inflammation in the GC-TME.

Macrophages are the most abundant inflammatory cells in TME. They exhibit significant plasticity and can freely switch from one phenotype to another; however, this depends on the signals received from their surrounding microenvironment [11, 12]. The process is called macrophage polarization. Based on the activation state, macrophages can be classified into classically activated macrophages (M1 macrophages) and alternatively activated macrophages (M2 macrophages) [13]. Although this dichotomy is somewhat arbitrary, it remains the most popular macrophage definition [14, 15]. It is generally believed that the M1 type has an anti-tumor property, whereas the M2 type is a pro-cancer factor. Also, the cancer cell is an “educator” that converts M1 to M2 [16]. M2 macrophages promote tumor metastasis and angiogenesis by secreting various cytokines and exerting immunosuppressive effects [17]. Macrophages recruited into TME by cytokines are referred to as tumor-associated macrophages (TAMs) [18]. TAMs predominantly exhibit an M2 phenotype, indicating a poor prognosis of solid tumors in TME [19, 20]. In particular, M2 macrophages could promote GC peritoneal metastasis [21]; extracellular vesicles secreted by M2 macrophages promoted GC progression [22]. Correspondingly, hypoxia GC cells induced the M2-type polarization in the TME [23]. The complex interaction mechanism of the two cells in the TME remains to be explored. Since hypoxia could promote immune evasion, angiogenesis, proliferation, and metastasis, research suggests that it can as well facilitate TAMs in tumor development. Additionally, the TAMs in tumor hypoxic regions mediate treatment resistance and promote cancer recurrence [24].

This paper focuses on the effect of hypoxia on macrophages then analyzes the role of hypoxic microenvironment and inflammatory microenvironment in GC development. *CXCL8* (C-X-C Motif Chemokine Ligand 8, also called *IL-8*) was overexpressed in hypoxic macrophages. Moreover, we discovered that the macrophage-derived *CXCL8* could activate the *JAK/STAT1* signaling pathway and promote GC invasion as well as proliferation. Besides, as a transcription factor, *STAT1* could upregulate *IL-10* (Interleukin 10) expression in GC; the latter could naturally trigger macrophage M2-type polarization. The M2 macrophages increased the release of *CXCL8*. The hypoxia-activated positive feedback loop *CXCL8/CXCR1/2* (*C-X-C* Motif Chemokine Receptor 1/2)/*STAT1/IL-10/NFKB1/CXCL8* could cascade and amplify the interaction between GC and macrophages leading to uncontrolled progressive signaling in GC.

## Materials and methods

### Bioinformatics

Hypoxic and normoxic cultured human monocyte-derived macrophage dataset GSE4630 was downloaded from the GEO (Gene Expression Omnibus) database (https://www.ncbi.nlm.nih.gov/geo/). They included two hypoxic-cultured samples and two normoxic-cultured samples. The GC RNA sequencing data were downloaded from the TCGA (The Cancer Genome Atlas, TCGA-STAD, 375 tumor samples vs. 31 normal samples, https://portal.gdc.cancer.gov/). CIBERSORT was used to determine the relative frequencies of immune cells in each sample. The GC and normal sample microarray data were also obtained from the GEO (GSE54129, 111 tumor samples vs. 21 normal samples). The differentially expressed genes were identified by the “limma” package (*P* < 0.05, and |FoldChange| ≥ 2). JASPAR (http://jaspar.genereg.net/) and UCSC (http://genome.ucsc.edu/) were performed to predict the transcription factor.

### Cell culture and human PBMCs isolation

**Fig. 1:**
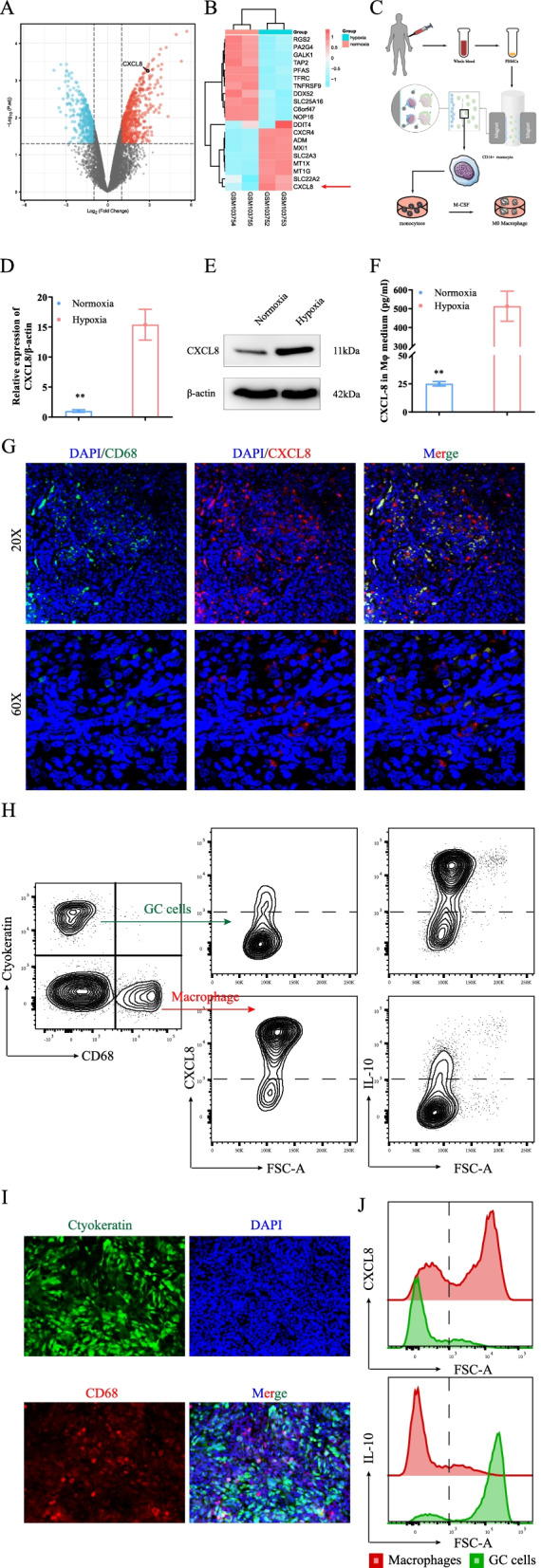
Hypoxia promoted macrophage-derived CXCL8 secretion. **A**, **B** Differential genetic analysis of the transcription data of macrophages cultured under hypoxia and normoxia showed that hypoxia could promote *CXCL8* expression. (A. volcano; B. heatmap). **C** MACS obtained human CD14^+^ monocytes and induced them into macrophages through M-CSF. **D**, **E** Hypoxia promoted human-derived macrophage CXCL8 expression at both transcription and protein levels. **F**. Hypoxia promoted the secretion of macrophage-derived cytokine *CXCL8* secretion. **G** An evident co-localization of macrophages and *CXCL8* in human GC tissue specimens. **H** Schematic of gating strategy of flow cytometry analysis. GC tissue were dissociated to obtain a single-cell suspension and stained with antibodies. Cells were first gated to exclude debris and dead cells (Sup Fig. 1D), then GC cells and macrophages were selected. Cells were further gated by cluster of *CXCL8* expression. **I** GC cells (green) and macrophages (red) were co-cultured under hypoxia to mimics the microenvironment. **J** *CXCL8* was mainly derived from macrophages and *IL-10* was expressed by GC cells in the co-culture system

The GC (HGC-27, NUGC3) cells, human normal gastric epithelial cell line (GES-1) were acquired from China Medical University (Shenyang, China). Mouse GC cancer cell (GAN-KP cells) was acquired from International Research Center for Medical Sciences, Kumamoto University [25]. Peripheral blood mononuclear cells (PBMCs) were obtained from healthy donors in Liaoning Cancer Hospital & Institute. CD14^+^ cells were enriched by depleting CD8^+^, CD19^+^, CD56+ and CD14+ cells by MACS (magnetic-activated cell sorting, EasySep, STEMCELL Technologies, 19,059) columns with negative selection. PBMCs were differentiated into immature macrophages (M0 macrophages) using 50 ng/mL human macrophage colony-stimulating factor (hM-CSF, Sigma, 81,627–83-0) for 7 days (Fig. 1C). All cells were cultured in RPMI-1640 (FUJIFILM Wako Pure Chemical Corporation, 189–02145) supplemented with 10% fetal bovine serum (FBS, Gibco 26,140–079) under 1% O_2_ or 20% O_2_ conditions as described previously [8, 10, 26]. In the co-culture model, macrophages and GC cells were added in a 1:1 ratio into a 10 cm dish (1 × 10^6^ cells for each) and analyzed after 7 days of co-culture.

### GC tissues and ethical approval

Between January 2009 and December 2012, gastric cancer and adjacent non-cancerous tissue samples were obtained from 526 patients subjected to adequate complete surgical resection (R0) of locally advanced GC surgical resection in Liaoning Cancer Hospital & Institute. All recruited patients had not received preoperative chemotherapy or radiotherapy. They signed a written informed consent before surgery. The follow-up of patients was closed on December 31, 2020. The study was approved by the Ethics Committee of Liaoning Cancer Hospital and Research Institute (20181226).

### Immunohistochemistry

The immunohistochemistry (IHC) was performed based on previously published protocols [27]. Immunohistochemistry was scored based on the intensity of staining and the proportion of positive cells. The blinded review was performed by two pathologists. *Ki67* (1:1000, Abcam, ab15580), *Caspase-3* (1:1000, Abcam, ab184787) *CXCL8* (5 μg/mL, R&D Systems, AF-208-NA).

### Immunofluorescence

The slices were overnight incubated with primary antibody at 4 °C. Then, they were incubated with species-appropriate rabbit/mouse secondary antibodies coupled with AlexaFluor dyes (488, 594, 1:200, Invitrogen, A32814, A32723, A32740), DAPI (1:1000, Dojindo, KT013) at room temperature for 1 h. *CXCL8* (5 μg/mL, R&D Systems, AF-208-NA), *CD68*, *CD163*, *CD86* (1:200, Abcam, ab213363, ab182422, ab270719). Olympus Fluoview FL1200 confocal microscope was used to capture 20x and 60x images.

### ELISA test

ELISA assay was used to measure the *CXCL8* in the supernatant of macrophages cultured in hypoxic or normoxic conditions and *IL-10* in the medium of GC cells. The procedure followed the instructions of the Human *CXCL8* kit (R&D Systems, D8000C) and Human IL-10 ELISA Kit (Abcam, ab185986).

### qRT-PCR

The quantitative real-time polymerase chain reaction (qRT-PCR) was performed as previously documented [8]. Primer sequences were designed by Sangon (China, Supplementary Table 1).

### Western blot analysis

The RIPA lysis buffer was used to extract total proteins from cells and tissues (Beyotime, Shanghai, China). Then, 10% SDS-PAGE gel was used to separate the proteins and transferred using the PVDF membrane. The membranes were overnight incubated with the primary antibodies (Supplementary Table 2) at 4 °C. Subsequently, they were co-cultured with the secondary antibody for 1 h. The ECL system (Amersham Imager 600) and ImageJ software were applied to observe and calculate grayscale values [8].

### Chromatin immunoprecipitation (ChIP)

ChIP enzymatic chromatin IP kit protocol (Cell Signaling Technology, China) was used to perform the ChIP assay, where 1 × 10^7^ logarithmic phase cells were subjected to ChIP. The chromatin was immunoprecipitated with an anti-STAT1 (Santa Cruz Biotechnology, USA, 1:50) and mouse IgG (Santa Cruz Biotechnology, USA, 1:100) on rotators at 4 °C for 16 h.

### Promoter-luciferase reporter assay

Promoter-Luciferase reporter assay confirmed the combination of *STAT1* and *IL-10* promoter sequence, *NFKB1*, and *CXCL8* promoter sequence, respectively. The recombinant *pGL-3* basic-plasmid contained truncated human *IL-10* (or *CXCL8*) promoter (wild type, wt, − 2000 ~ + 99) or mutant *IL-10* (or *CXCL8*) promoter (mutant type, mut). The plasmids were transfected with the POLO3000 transfection reagent (Research and science, China). After 48 h, the luciferase activity of each group was evaluated following the manufacturer’s instructions.

### Colony formation assay

A total of 1 × 10^5^ GC cells/each group were seeded into 6-well plates for 2 weeks. The colonies were washed three times using PBS, fixed with 4% paraformaldehyde, and stained with Diff-Quik III Kit (Muto Chemical, ZS0003). The stained colonies were observed under a light microscope (IX81 Olympus).

### Cell counting assay

Cell proliferation was evaluated using the Cell counting kit (Cell counting kit 8, CCK-8). The cells were incubated at 37 °C for 24, 48, 72, and 96 h. Subsequently, the medium was discarded then the chromogenic solution at 10:1 was prepared. For incubation, a color-developing solution (10 μl) was added to the 96 well plates at 37 °C for 2 h. The optical density (OD) was detected using the UV spectrophotometer (BioTek Synergy H1) at 450 nm.

### Transwell

Gastric cancer cells were seeded in Transwell upper chambers coated with gelatin. The lower chambers with 600 μL RPMI-1640 with 20% FBS. After 24 h, the cells were fixed and stained using methanol, hematoxylin, and eosin (Sigma-Aldrich, St. Louis, MO, USA). The upper chamber was removed and the lower layer migrating cells counted under the microscope (IX81 Olympus).

### Real-time imaging of cell migration

A total of 200 μl Matrigel (BD Biosciences, 356,235) pre-coated the bottom of the six-well plates, the cells were then inoculated for 24 h. Thereafter, a 6-well plate was cultured and imaged using KEYENCE BZ-X700 (KEYENCE, Japan), equipped with CO_2_ and temperature control chamber as well as a time-lapse tracking system. BZ-X Viewer software (KEYENCE) was used to capture phase-contrast images at intervals of every 10 min for 48 h, while the BZ-X Analyzer software (KEYENCE) was used to convert the continuous images into movie files. Furthermore, KEYENCE video editing and analysis software was used to analyze cell migration in the movies. Microsoft Excel 2010 was used to process the trace data to create XY coordinate graphs and distance measurements.

### Flow cytometers

The cell concentration was adjusted to 1 × 10^6^ cells/ml in PBS containing 2% FBS. The cell suspensions were incubated with antibodies (BioLegend, Cat# 137005, Cat# 333805, Cat# 506804, Cat# 505007; abcam, ab289967, ab52460) for 30 min on ice, washed with PBS containing 2% FBS, centrifuged twice, and suspended in PBS. Flow cytometry was performed with a FACSVerse instrument (BD Biosciences). The flow cytometry data were analyzed using FlowJo 3.3 software (Tree Star).

### Xenograft mouse model

For subcutaneous tumor xenografts, an estimated 1 × 10^6^ mouse GC cells/0.2 ml PBS (with or without CXCL8 which injected every 3 days) were subcutaneously injected into the right axillary region of a 5-week-old female BALB/ C mouse. After 28 days of injection, tumors were collected, measured volumes weighed, and photographed. The volume was calculated using the following formula (tumor volume = L*W*W/2).

For the intraperitoneally model, approximately 5 × 10^6^ GC cells/0.1 ml PBS (with or without CXCL8) were injected into the mouse as mentioned above. Similarly, the mice were euthanized 28 days after injection, then the volume of ascites and the number of visible (> 0.1 cm) metastatic nodules in the peritoneal cavity were measured.

### Other reagent or resource

Recombinant Human *IL-8* (CXCL8, PeproTech, 200–08), Recombinant Human *IL-10* (PeproTech, 200–10), Recombinant Human M-CSF (PeproTech, 300–25), Repertaxin (Sigma-Aldrich, 266,359), Fludarabine (Tocris, 3495/10), Sarsasapogenin (Selleck, S3607), Bay11–7082 (Selleck, S2913), Anemoside B4 (Selleck, S9081).

### Statistical analysis

GraphPad Prism 9.0 (GraphPad Software Inc) and SPSS 24.0 statistical software (IBM) were used for statistical analyses of data. The student’s *t*-test was utilized to perform statistical analysis. *P* < 0.05 was considered statistically significant.

## Results

### Hypoxia promotes macrophage-derived *CXCL8* secretion

Relevant expression profile data were downloaded from the GEO database (GSE4630) to identify the reflection of macrophages under hypoxic conditions. The dataset included four PBMCs-derived macrophages samples. Two of them were cultured in 0% O_2_, while the other two were cultured in 20% O_2_. The “limma” package was used to identify the differentially expressed genes (DEGs, Supplementary Table 3). As a type of immune cell, macrophages could disrupt the microenvironment through cytokines. Thus, this work assessed the effect of hypoxia on cytokine-related gene expression. Among them, *CXCL8* was significantly upregulated in the hypoxic-cultured macrophage (Fig. 1A, B). This implied that hypoxic TME might stimulate *CXCL8* secretion by macrophages; nevertheless, this warrants further validation. CD14^+^ positive monocytes by MACS (magnetic-activated cell sorting) negative selection were obtained from healthy volunteers to accurately reflect the in vivo features of macrophages. The PBMCs were induced into immature macrophages (M0 macrophages) for 7 days by *M-CSF* (Fig. 1C). Subsequently, incubation of M0 for 72 h was continued under hypoxic (1% O_2_) and normoxic (20% O_2_) conditions, respectively. Both qRT-PCR and western-blot showed that *CXCL8* was overexpressed in hypoxic-cultured M0 (Fig. 1D, E). Similarly, the upregulated *CXCL8* in the supernatant of hypoxic-culture M0 was confirmed through the ELISA test (Fig. 1F).

On the other hand, we also examined the effect of hypoxia on GC-derived *CXCL8* expression. We found that hypoxia slightly promoted the expression and release of *CXCL8* in GCs, but not as dramatically as in macrophages (Sup Fig. 1A, B). At the same time, we reviewed the microarray data obtained in previous studies [8]. The data indicated that the knockdown of HIF-1α had no effect on the expression of *CXCL8* in GC cells (Sup Fig. 1C). Immunofluorescence analysis in human GC tissues showed extensive co-localization of *CD68* and *CXCL8*, suggesting that macrophages could secrete *CXCL8* (Fig. 1G). To determine the cells responsible for *CXCL8* and IL-10 production in GC tissue, we examined *CXCL8*-expressing cells by flow cytometry (Sup Fig. 1D, Fig. 1H). We observed that *CXCL8*-expressing cells were expressed of predominantly macrophage (CD68^+^, Fig. 1H, middle, down), but not GC cells (Ctyokeratin^+^, Fig. 1H, middle, up). Subsequently, we co-cultured GC cells and macrophages under hypoxic conditions (Fig. 1I). *CXCL8* was mainly derived from macrophages in vitro (Fig. 1J, up; Red: macrophage; Green: GC cells).

### *CXCL8* correlates with poor prognosis and tumor progression of GC

**Fig. 2:**
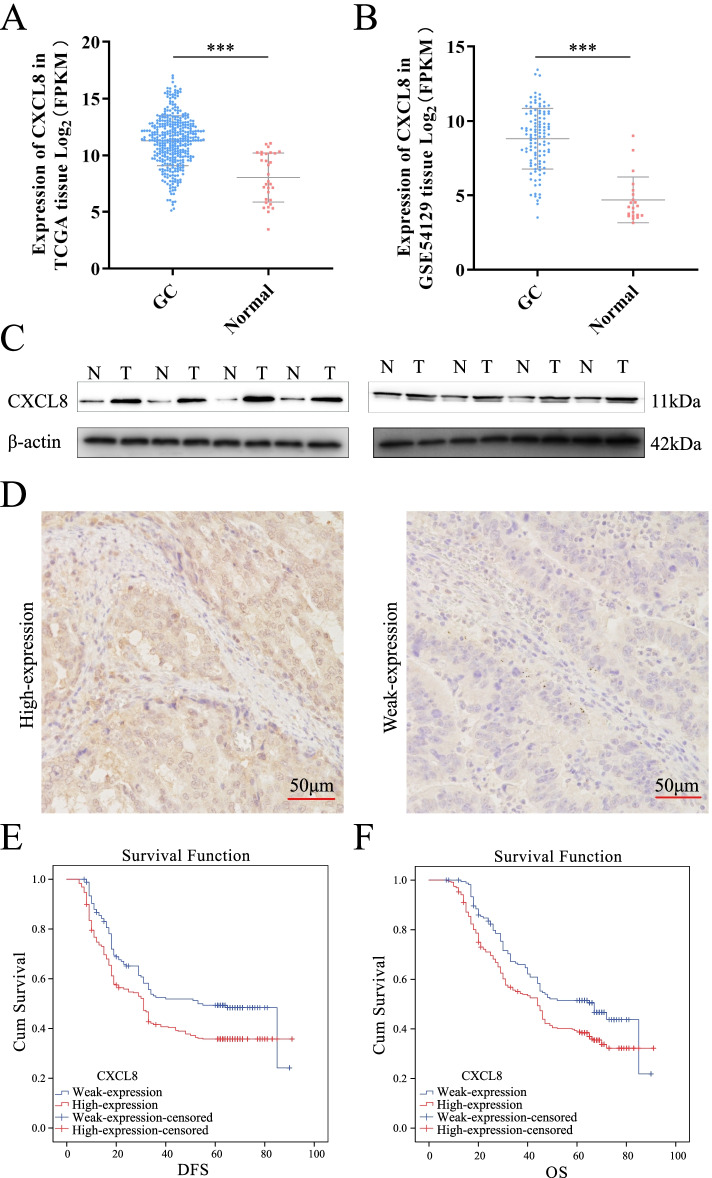
*CXCL8* overexpression in GC promotes poor survival. **A**, **B** *CXCL8* was up-regulated in GC tissue both in TCGA-GC and GEO-GC cohort. **C** In matched GC and adjacent tissues, *CXCL8* was highly expressed in cancer tissues. **D** Representative image of *CXCL8* expression in GC tissue. (Left: high-expression; Right: weak-expression). **E**, **F** *CXCL8* promoted poor DFS and OS in the GC cohort. (E. DFS; F. OS)

*CXCL8* expression in TCGA and GEO (GSE54129) was assessed to further elucidate its role in GC. *CXCL8* expression was significantly upregulated in GC tissues compared to that in normal tissues (Fig. 2A, B). The CIBERSORT analysis showed that the expression of *CXCL8* was positively correlated with macrophage infiltration in GC-TME (Sup Fige. 1E). Further, the samples were divided into *CXCL8*-high group and *CXCL8*-weak group according to the median of *CXCL8* expression. The infiltration ratio of macrophages in the TME in the *CXCL8*-high group was significantly higher than that in the *CXCL8*-weak group (Sup Fig. 1F). Subsequently, we assessed the clinical significance of *CXCL8* based on the Liaoning cancer hospital cohort. *CXCL8* in cancerous tissues was remarkably higher than that in paired paracancerous tissues (Fig. 2C). Figure 2D reveals the representative images of high expression and low expression *CXCL8* in GC tissue. Moreover, high *CXCL8* expression promoted poor DFS (disease-free survival, Fig. 2E) and OS (overall survival, Fig. 2F). The cohort information is listed in Supplementary Table 4.

### *CXCL8* activates the *CXCL8-CXCR1/2* axis of GC and exacerbates the malignant phenotype

**Fig. 3:**
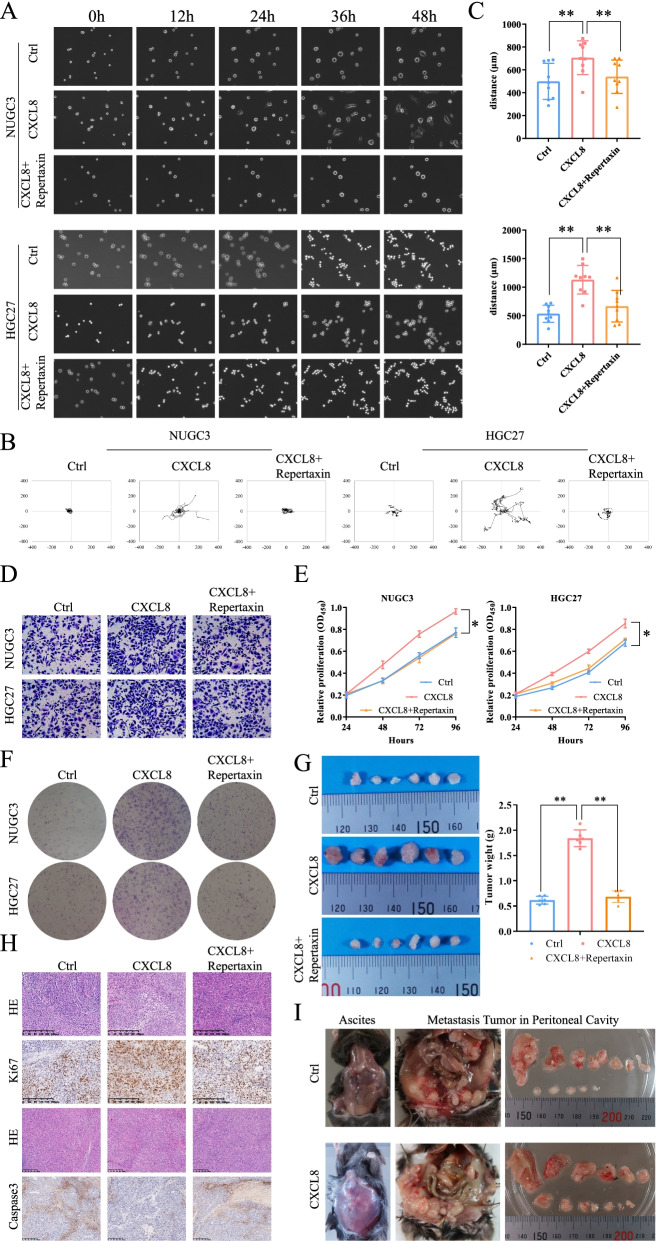
*CXCL8*-*CXCR1/2* deteriorates the malignant phenotype of GC. **A** Migration patterns of GC cells on Matrigel compared to cells cultured with *CXCL8* and/or *CXCL8-CXCR1/2* inhibitor repertaxin. **B** Migration trail of GC cells cultured with *CXCL8* or *CXCL8* and *CXCL8-CXCR1/2* inhibitor. **C** Migration average distances of GC cells in each group. **D** Transwell exhibited the differences in the number of permeable cells in each group. **E**, **F** CCK-8, and colony formation assay confirmed *CXCL8* could promote GC proliferation which could be inhibited by *CXCL8-CXCR1/2* inhibitor. **G** *CXCL8* increased the subcutaneous tumor size and weight. **H** IHC representative image of subcutaneous tumors showing that *CXCL8* could promote *Ki67* and inhibit the expression of *Capcase3*; the inhibitors could counteract this effect. **I** *CXCL8* induced ascites formation and intra- metastasis of GC

In vitro and in vivo assays were performed to assess the function of *CXCL8* on GC cells. The GC cells were seeded on the six-well plates coated with Matrigel. The real-time imaging recorded the motion state of the cells. Migration of GC cells was significantly improved when co-culture with recombinant human *CXCL8*. *CXCL8* accelerated cells migration (50 ng/ml, Fig. 3A-C, Sup movie 1, 2). Nonetheless, the enhanced motility was inhibited by adding *CXCL8-CXCR1/2* inhibitors (Repertaxin, 50 μg/ml, Fig. 3A-C, Sup movie 1, 2). The promoting impact of *CXCL8* on GC invasion ability was achieved by the *CXCL8-CXCR1/2* axis. In keeping with this result, the Transwell assay also exhibiting *CXCL8* caused an increase in the number of cells penetrating the membrane, but was blocked by repertaxin (Fig. 3D). On cell proliferation, the CCK-8 and colony formation assay demonstrated the promotion of cell proliferation by *CXCL8* (Fig. 3E, F); this was also achieved through the *CXCL8-CXCR1/2* axis (Fig. 3E, F). Likewise, we knockdown the expression of *CXCR1/2* in GC cells (Sup Fig. 2A, B). After inhibited the expression of *CXCR1/2*, the promoting effect of *CXCL8* on cell motility was significantly attenuated (Sup Fig. 2C, D, Sup movie 3, 4). The number of invasive cells in the Transwell assay also decreased accordingly (Sup Fig. 2E). Colony formation and CCK-8 assays also confirmed that with the deletion of *CXCR1/2*, *CXCL8* had almost no effect on cell proliferation (Sup Fig. 2F-H).

In the in vivo assays, we confirmed that *CXCL8* promoted the formation of the subcutaneous tumor. After injection of mouse-GC cells stimulated by *CXCL8*, the subcutaneous tumors had a larger size and heavier weight than the control. In contrast, no significant difference was noted in tumor size between the control and the inhibitor-added groups (Fig. 3G). At the same time, the expression of Ki67 increased and the expression of Caspase3 decreased in the *CXCL8* group; after adding the inhibitor, Ki67 decreased and Caspase3 increased (Fig. 3H). This implied that *CXCL8* could promote GC malignant phenotype; this was achieved through the *CXCL8-CXCR1/2* axis. Furthermore, unlike other digestive system cancers metastasizing primarily through the vasculature, GC cells had a greater tendency to develop peritoneal metastases. Therefore, we evaluated the effect of *CXCL8* on these types of metastases. After intraperitoneal GC cells co-culturing with *CXCL8*, the mice evolved peritoneal metastases (Fig. 3I).

### *JAK/STAT1* pathway is downstream of *CXCL8*

**Fig. 4:**
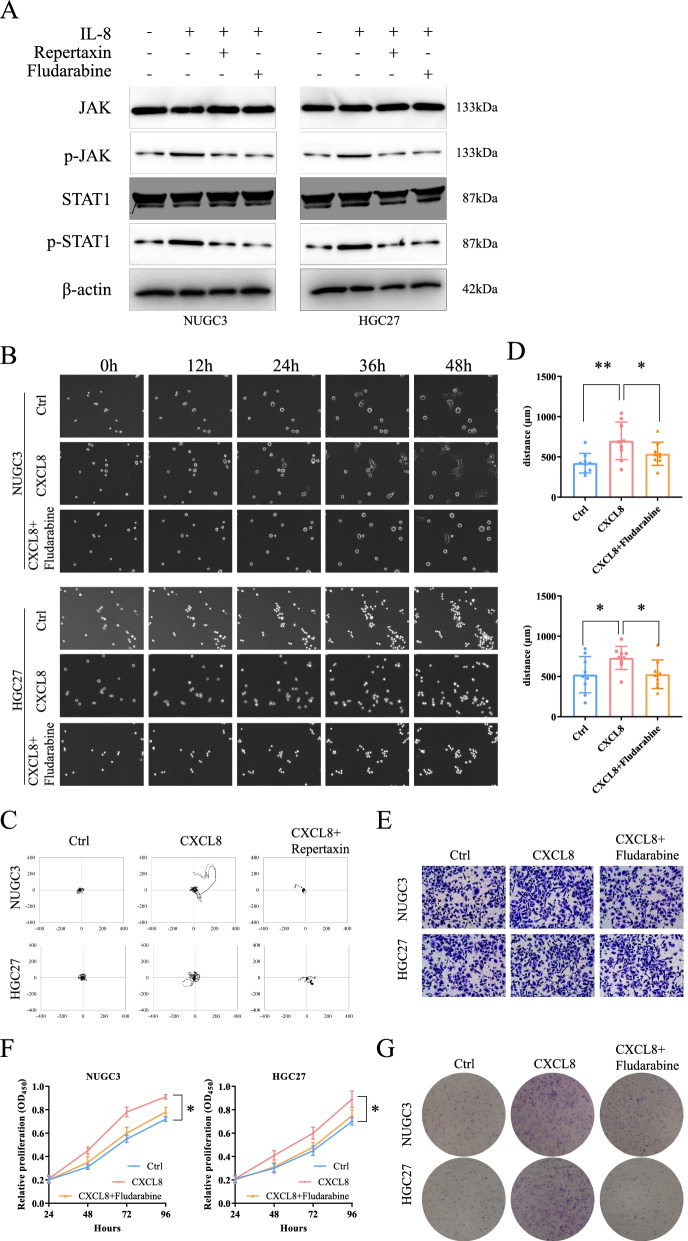
*CXCL8-CXCR1/2* activated *JAK/STAT1* signal pathway and promoted GC progression. **A** *CXCL8* activated the *JAK/STAT1* signal pathway. The *STAT1* specific inhibitor (Fludarabine) could antagonize the effects of *CXCL8*. **B**-**D** Migration patterns, migration trail, and average traveled distances of GC cells treated with *CXCL8* or *CXCL8* and Fludarabine together. **E** Fludarabine limited the GC invasion. **F**, **G** CCK-8, and colony formation assay revealed that the Fludarabine could resist *CXCL8’s* promotion of GC proliferation

After clarifying the oncogenic effect of *CXCL8* on GC, it is essential to explore the possible mechanisms involved. Since *JAK/STAT1* pathway is a canonical downstream of the chemokine signaling pathway [28, 29], the effect of the *CXCL8-CXCR1/2* axis on the *JAK/STAT1* pathway activation was assessed. The protein expression of *p-JAK*, *p-STAT1* was significantly upregulated after stimulation of GC with *CXCL8*, while the expression of *JAK*, *STAT1* did not significantly change (Fig. 4A). Similarly, the *CXCL8-CXCR1/2* inhibitors, repertaxin, inhibited the activation of the *JAK/STAT1* pathway by *CXCL8* (Fig. 4A). *JAK/STAT1* pathway might be the downstream effector of the *CXCL8-CXCR1/2* axis. To validate the result, *CXCL8*-induced GC cells were co-cultured with *STAT1* specific inhibitor (Fludarabine). As expected, the activation effect of *CXCL8* on *JAK/STAT1* disappeared when cells were treated with Fludarabine (50 μM, Fig. 4A). This confirmed that the *JAK/STAT1* is the downstream effector of *CXCL8*. Unsurprisingly, the *JAK/STAT1* pathway inhibitors also limited the effects of *CXCL8* on invasion (Fig. 4B-E, Sup movie 5, 6) and proliferation (Fig. 4F, G). Thus, a preliminary conclusion would be exogenous *CXCL8* continuity activated the *CXCL8/CXCR1/2-JAK/STAT1* pathway and triggered invasion and proliferation of GC.

### *STAT1* regulates the expression of *IL-10* directly but not *HIF-1α*

**Fig. 5:**
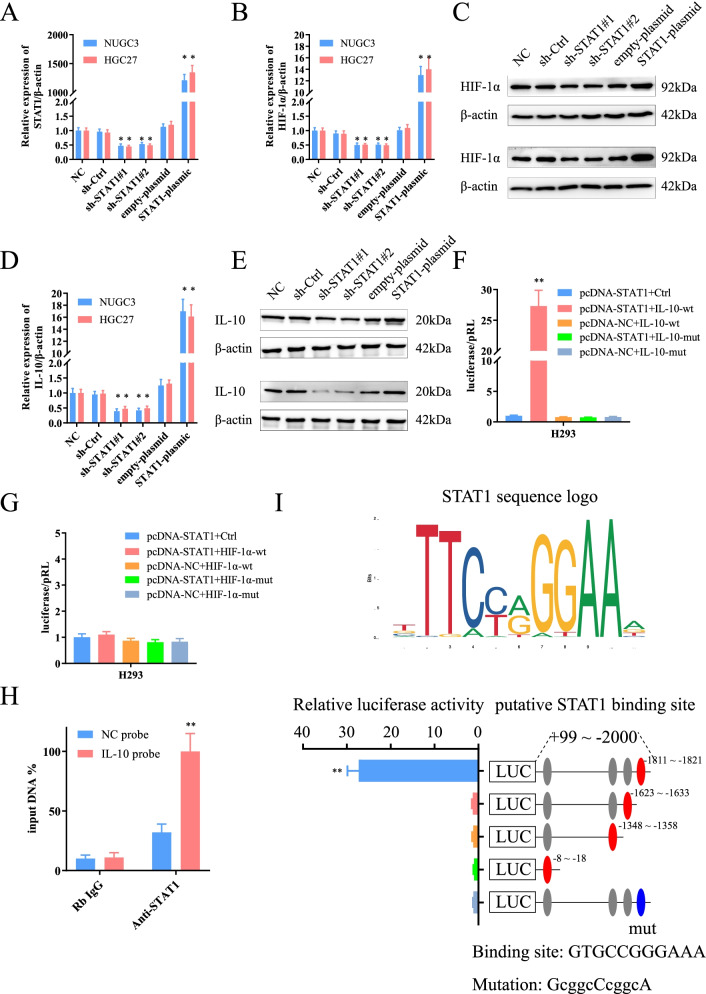
Transcription factor *STAT1* could directly upregulate *IL-10* expression. **A** The shRNA and plasmic regulated *STAT1* expression. **B**, **C** *STAT1* regulated *HIF-1α* expression at transcription and protein levels. **D**, **E** *STAT1* could also influence *IL-10* expression at transcription and protein levels. **F**, **G** The promoter-luciferase reporter assay, showing that *STAT1* directly promoted *IL-10* expression but not *HIF-1α*. **H** The interaction of *STAT1* with *IL-10* shown using ChIP assays with control (IgG) or anti-*STAT1* antibody. **I** Deletion and selective mutation analysis identified *STAT1*-responsive regions in the *IL-10* promoter. Luciferase reporter plasmids containing serially truncated or mutated *IL-10* promoter constructs were co-transfected with OE-*STAT1* into NUGC3 cells, and relative luciferase activities were detected. The − 1811 ~ − 1821 sequence was the binding site

*STAT1* belongs to the *STAT* protein family that forms homo- or hetero-dimers and translocates to the nucleus to exert regulatory effects as a transcriptional activator. Naturally, we sought to identify the downstream regulatory molecules of *STAT1*. Through the JAPAR database, the target genes of *STAT1* were predicted. Among them, *HIF-1α* and *IL-10* attracted our attention. The former was the hypoxic associated core effector molecule, while the latter was closely related to the polarization of macrophages. It should assess the influence of *STAT1* on *HIF-1α* and *IL-10*. The shRNA and OE-vector of *STAT1* could up-or down-regulated *STAT1* expression (Fig. 5A). *STAT1* inhibition was followed by a downregulation in *HIF-1α* and *IL-10* expressions both at the transcriptional and protein levels (Fig. 5B-E). In contrast, both were upregulated with *STAT1* overexpression (Fig. 5B-E). With the promising results, luciferase reporter assay and ChIP were performed to assess the interaction of *STAT1* with *HIF-1α* and *IL-10* promoters. Therefore, full-length *HIF-1α* and *IL-10* promoters were cloned into luciferase reporter plasmids, respectively. The luciferase reporter assay revealed that the up-regulated *STAT1* could enhance the luciferase activity of *IL-10-wt*, but not *IL-10-mut* (Fig. 5F). Unfortunately, *STAT1* did not affect the fluorescence intensity of *HIF-1α*, whether *HIF-1α-wt* or *HIF-1α-mut* (Fig. 5G). This suggestd that as a transcription factor, *STAT1* could directly activate the transcription of *IL-10*, however, *HIF-1α* promotion might rely on other indirect pathways. Subsequently, the ChIP assay confirmed that the *STAT1* antibody could dismantle the *IL-10* sequence but not the control IgG (Fig. 5H). A series of reporter genes comprising truncated *IL-10* promoter sequences were constructed to further refine the target sequence of the promoter. The luciferase reporter assay showed that deleting the region between − 1811 and − 1821 bases of *IL-10* promoter severely abolished *IL-10* by *STAT1* activation (Fig. 5I).

Unsurprisingly, *CXCL8* could significantly promote the expression of *IL-10* in GCs, but it did not alter *HIF-1α* expression significantly (Sup Fig. 3A, B). Following the increase of *IL-10* in the TME, we further assessed the effect of *IL-10* and hypoxia on the *JAK/STAT1* pathway. Consistent with previous studies [30], both *IL-10* and hypoxia were also activators of the *JAK/STAT1* pathway (Sup Fig. 3C, D). That was to say, *CXCL8* overexpressed due to hypoxia could promote the expression of *IL-10* together with the hypoxic microenvironment, and the *IL-10* could amplify this effect through positive feedback (Fig. 7). Further, we also compared the differences in the effects of hypoxic microenvironment and *CXCL8* on *IL-10* expression. The results showed that both tumor hypoxia and *CXCL8* could increase the expression of *IL-10* in GC, but the gain effect of *CXCL8* was more significant (Sup Fig. 3E).*CXCL8* remained the dominant factor in inducing *IL-10* expression. In addition, both of in vitro and in vivo analysis confirmed that *IL-10* was mainly expressed in GC cells (Fig. 1H, right, up; Fig. 1J, down). In subcutaneous tumors, the tumor size (Sup Fig. 3F), the expression of *IL-10* and *CXCL8* also decreased with the addition of Repertaxin (Sup Fig. 3G, H). The *CXCL8/CXCR1/2-JAK/STAT1/IL-10* axis was an indispensable oncogenic factor in the TME.

### *IL-10* induces M2 polarization through the *NF-κB* pathway and promotes *CXCL8* expression

**Fig. 6:**
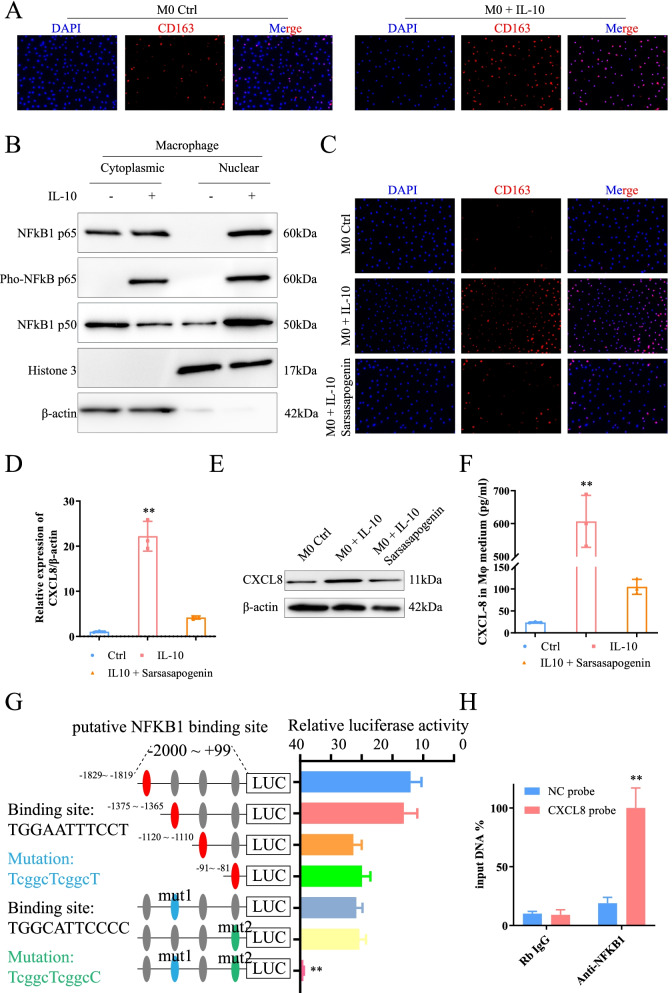
Cytokines *IL-10* positive feedback induces macrophage M2 polarization and upregulates macrophage-derived *CXCL8* expression. **A** *IL-10* promoted macrophages *CD163* expression and induced M2 polarization. **B** *IL-10* activated *NF-κB* signaling pathway. **C** The *NF-κB* pathway inhibitor (Sarsasapogenin) could limit *IL-10* induced M2 polarization. **D** *E. IL-10* upregulated *CXCL8* expression, and it could be prevented by *NF-κB* pathway inhibitor. **F**. *IL-10* could promote *CXCL8* secretion, and Sarsasapogenin could antagonize the effect of *IL-10*. **G** Deletion and selective mutation analysis recognized the *NFKB1*-responsive regions in the *CXCL8* promoter. Luciferase reporter plasmids containing serially truncated or mutated *CXCL8* promoter constructs were co-transfected with *OE-NFKB1* into macrophage cells, and relative luciferase activities were detected. The − 1829 ~ − 1819 and − 91 ~ − 81 sequences were all the binding sites

As evidenced above, hypoxic-macrophage-derived *CXCL8* could promote *IL-10* expression by activating *JAK/STTA1* in GC. Besides, studies have shown that cytokine *IL-10* can induce M2 polarization [31]. Therefore, the effect of *IL-10* on macrophages and the potential underlying mechanisms should be confirmed. Unsurprisingly, immunofluorescence confirmed that *IL-10* significantly promoted *CD163* expression in PBMCs induced macrophage, and M2 polarization (Fig. 6A). Abnormally activated *NF-κB* pathway exists in TAMs [32]. Thus, it is necessary to evaluate the effect of *IL-10* on the *NF-κB* pathway in macrophages. The addition of *IL-10* upregulated p50 and p65 expression in the nucleus and promoted p65 phosphorylation (Fig. 6B). Subsequently, *IL-10* and BAY 11–7082 (10 μM) or Sarsasapogenin (50 μM) were used to inhibit the *NF-κB* pathway and co-cultured macrophages. Sarsasapogenin, a steroidal sapogenin, is purified from the Chinese Materia Medica Anemarrhena asphodeloides Bunge (rhizome) [33]. It can provoke the generation of reactive oxygen species and activate unfolded protein response (UPR) signaling pathways. It specifically targeting *IκB*, potently inhibits *NF-κB* activation [34]. Both sarsasapogenin and Bay11–7082 attenuated *CD163* expression (Fig. 6C, Sup Fig. 4A). It inhibited the M2 polarization. This demonstrates that the effect of *IL-10* on macrophage M2 polarization was partly achieved through the *NF-κB* pathway. This implied that the *IL-10-NF-κB* axis was implicated in *CXCL8* regulation at the transcriptional level. Besides, we analyzed the effects of *IL-10* and *NF-κB* pathways on *CXCL8* expression. *IL-10* upregulated *CXCL8* expression at the transcriptional and protein levels (Fig. 6D, E) and intensified the secretion of the latter (Fig. 6F). Correspondingly, the *NF-κB* pathway could antagonize this effect (Fig. 6D-F, Sup Fig. 4B-D). Subsequently, we examined the effects of *CXCL8* and hypoxia on macrophage polarization, *IL-4* was set as a positive control. *CXCL8* had no significant effect on the M2 polarization of macrophages, and hypoxia could induce the M2 polarization, but the polarized level was not as intensity as that of *IL-4* (Sup Fig. 4E). In the macrophages and GC cells co-culture system, the expression of *CXCL8* decreased with *IL-10* inhibitor added (Sup Fig. 4F). In vivo assay, after inhibited the expression of *IL-10*, the tumor contracted (Sup Fig. 4G) and the expression of *CXCL8* in the tumor tissue also decreased (Sup Fig. 4H). With the inhibition of *IL-10* expression, the expression of macrophage-derived *CXCL8* in the tissue was also suppressed (Sup Fig. 4I).

The subsequent transcription factor luciferase reporter assay and ChIP confirmed our suspicions. The gene (*p50*) was bound to the promoter of *CXCL8* (Fig. 6G, H). A series of truncated *CXCL8* promoter sequences were constructed, which included at least one potential binding site (in red). Nevertheless, every sequence could promote *CXCL8* expression. Mutated the mut1 (in blue, − 1829 ~ − 1819) and mut2 (in green, − 91 ~ − 81) sequences simultaneously, the fluorescence intensity significantly decreased. This confirmed the presence of two *NFKB1* binding sites in the promoter sequence and up-regulated *CXCL8* expression. In this way, a positive feedback loop was formed between macrophage and GC cells that exacerbated tumor deterioration under the catalysis of hypoxic TME.

## Discussion

TME of most solid tumors is characterized by hypoxia and inflammation [35]. The crosstalk between TME and cancer cells promotes tumor growth and progression [36]. Cytokines are the critical bridge that maintains this intercellular communication; besides, they can favorably shape the TME for tumor cells [37]. Gastric cancer cells rely on this complex environment to achieve sustainable development, invasion, and metastasis. In our previous study, we continuously focused on the effect of hypoxia on the progression of GC cells [8]. Nevertheless, this effect on TME stromal cells and inflammatory cells has been overlooked.

Of note, tumor-infiltrating macrophages release the strongest signal in inflammatory TME [38]. Thus, we concentrated on the effect of hypoxia on macrophage cytokines i.e., the communication tools. Among them, *CXCL8* exhibited the most potent response to the hypoxic signal. *CXCL8* is one of the earliest and most comprehensively studied chemokines. It was discovered, purified, and sequenced in monocytes in the 1980s [39]. *CXCL8* is secreted by different cells, including monocytes, alveolar macrophages, fibroblasts, endothelial cells, and epithelial cells [40, 41]. Additionally, cytokines (interleukin-1, interleukin-6, *CXCL12*, *TNFα*), hypoxia, reactive oxygen species (ROS), bacterial particles could stimulate *CXCL8* expression. The transcription factor *NF-κB* and activator protein-1 (*AP-1*) were also implicated in *CXCL8* activation [42, 43]. Notably, *CXCL8* was nearly undetectable in unstimulated cells. In contrast, the above stimulation would upregulate *CXCL8* expression by 10 to 100 times [44]. Besides, this type of explosive growth provides a basis for its role in cancers. *CXCL8* binds G protein-coupled receptors or CXCR1/2 to transmit its signal. *CXCL8-CXCR1/2* axis stimulates endothelial cells and promotes tumor angiogenesis [45]. Blocked *CXCL8-CXCR2* signal transmission between TAMs and cancer cells could improve the effect of anti-PD1 treatment [46, 47]. This study found that the *CXCL/8-CXCR1/2* axis activated the *JAK/STAT1* pathway in GC, thereby promoting the malignant phenotype. Further, *STAT1* acted as a transcription factor directly regulating *IL-10* expression, this was closely associated with macrophages. In addition, it could not ignore that *STAT1* also affected the *HIF-1α* expression, which was the most crucial element for the organism responding to hypoxia. The indirect regulatory pattern of hypoxia/*CXCL8/CXCR1/2/JAK/STAT1/HIF-1α* might be a non-classical adaptation pattern of GC cells to the hypoxic microenvironment.

*IL-10* is one of the most famous molecules causing macrophage polarization [48]. It triggers M2 polarization by regulating the *NF-κB* and *JAK/STAT* pathway [49]. Generally speaking, *NF-κB* pathway activation is often regarded as a marker of M1 macrophages because of its pro-inflammatory effects. However, various tumor-associated cells appeare to exhibit complex characteristics as the TME forms. Even inflammatory responses are involved in tumor immunity while inducing maturation of tumor-associated fibroblasts [50]. TAMs also deviate from the traditional classification. Although the simple dichotomy of macrophage (M1 and M2) is widely used, researchers continue to express doubts. With the advancement of single-cell technology, further sub-classification of macrophage by markers on the surface of the macrophage membrane deepen the understanding of the TME [51]. Specifically, tissue-resident macrophages and monocyte-derived macrophages have entirely different biological functions, however, the two co-exist in TME [19]. The subclassification theory of M2 macrophages indicates that the M2d subtype is the closest to TAMs, and the activation of *NF-κB* pathway is also a sign event of M2d activation [52, 53]. This means that the activation of the *NF-κB* pathway in the complex TME is still a double-edged sword. Then, it was natural for us to further evaluate its effect on macrophage and macrophage-derived *CXCL8*. Our results demonstrated that *IL-10* regulated M2 polarization via the *NF-κB* pathway and affected *CXCL8* expression. Subsequent luciferase reporter assays and ChIP assays confirmed the regulatory role of the transcription factor *NFKB1* (*p50*) on *CXCL8*. This was also consistent with previous findings [54, 55]. Thus, macrophage-derived *CXCL8* could further promote the deterioration of GC and cause *IL-10* expression, recurrence of the cycle.

The above results illustrate a novel paradigm of GC-TAMs’ interaction: hypoxia spurs the release of overexpressed *CXCL8* by macrophages which then activates the *CXCL8/CXCR1/2-JAK/STAT1* pathway and promotes GC progression. Moreover, this pathway directly promotes the expression of GC-derived *IL-10*, further accelerating the release of macrophage-derived *CXCL8* under the *IL-10/NF-κB/CXCL8* axis. In this process, the M2-type polarization is simultaneously induced. This causes ordinary macrophages involved in immune surveillance to “betray” into TAMs implicated in immune escape and suppression. Hypoxia/*CXCL8/CXCR1/2-JAK/STAT1/IL-10/NF-κB/CXCL8* forms a positive feedback loop between GC and macrophage (or TAMs). As the core molecule of this circuit, *CXCL8* is a high-risk factor for GC prognosis. This suggests that the signaling loop is a potential therapeutic target. Also, research on anti-*CXCL-8* therapy in solid tumors recruits or collates data (NCT01831310, NCT02536469, NCT04347226), and we have reason to expect these corresponding results.

## Conclusion

**Fig. 7:**
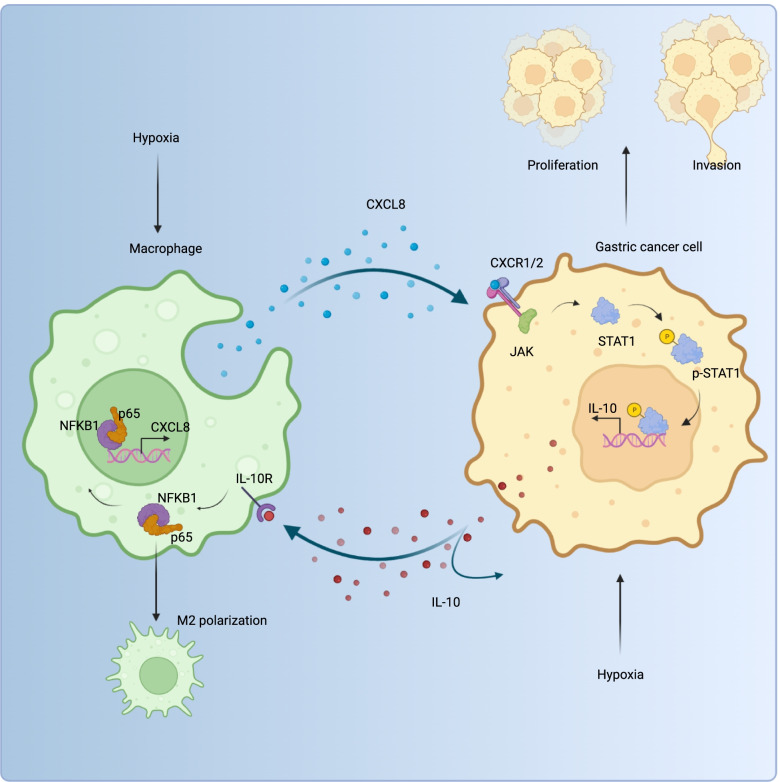
The mechanism graph of the positive feedback loop between GC cells and TAMs. Hypoxia-induced macrophage-derived *CXCL8* secretion activated *CXCL8- CXCR1/2-JAK/STAT1* signaling pathway and deteriorated the GC malignant phenotype. The transcription factor *STAT1* could directly promote *IL-10* expression. The latter activated the macrophage *IL-10/ NF-κB* signaling pathway and induced M2 polarization. The transcription factor *NFKB1* directly upregulated *CXCL8* expression

In conclusion, our work identifies a hypoxic-activated, inflammation-associated molecular network; which involve *CXCL8*, *CXCR1/2*, *JAK/STAT1*, *IL-10*, and *NF-κB* signaling pathways that regulate a positive feedback loop between GC cells and TAMs, tumor progression, macrophage polarization, as well as patient outcomes (Fig. 7). These results indicate the potential of this feedback loop as a therapeutic target for GC.

## Supplementary Information

**Additional file 1: Supplementary Table 1**. Primer sequence. **Supplementary Table 2**. Antibodies information.

**Additional file 2.**

**Additional file 3.**

**Additional file 4: Supplementary Fig. 1.** A. Hypoxia promoted *CXCL8* expression in GC cells at mRNA level. B. Hypoxia induced the secretion of GC-derived cytokine *CXCL8* secretion. C. The knockdown *HIF-1α* did not affect the expression of *CXCL8*. D. Cells were gated to exclude debris and dead cells. E. CXCL8 was positively correlated the proportion of infiltrating macrophages in TME. F. The infiltration ratio of macrophages in *CXCL8*-high group was significantly higher than that in *CXCL8*-weak group in TME. **Supplementary Fig. 2.** A, B. The siRNAs could effectively inhibit the expression of *CXCR1* and *CXCR2.* C. Migration patterns of GC cells on Matrigel compared to cells cultured with *CXCL8* alone and *CXCL8 + si-CXCR1/2*. D. Migration average distances of GC cells in each group. E. Transwell exhibited the differences in the number of permeable cells in each group. F, G, H. Colony formation assay and CCK-8 confirmed *CXCL8* could promote GC proliferation which could be inhibited by *si-CXCR1/2*. **Supplementary Fig. 3.** A. *CXCL8* promoted *IL-10* expression in vitro. B. *CXCL8* slightly promotes the expression of *HIF-1α*. C, D. *IL-10* and *hypoxia* activated the *JAK/STAT1* signal pathway. The *STAT1* specific inhibitor (Fludarabine) could antagonize the effects. E. *CXCL8* was more able to promote the expression of GC-derived *IL-10* than hypoxia. F. *CXCL8-CXCR1/2* inhibitor limited the growth of subcutaneous tumors. G, H. *CXCL8-CXCR1/2* inhibitor restricted the expression of CXCL8 and IL-10 in tumor tissues. **Supplementary Fig. 4.** A. The *NF-κB* pathway inhibitor (Bay11–7082) could limit *IL-10* induced M2 polarization. B, C. *IL-10* upregulated *CXCL8* expression, and it could be prevented by *NF-κB* pathway inhibitor (Bay11–7082). D. *IL-10* could promote *CXCL8* secretion, and Bay11–7082 could antagonize the effect of *IL-10*. E. *CXCL8* and hypoxia could slightly induce macrophage M2 polarization. F. *IL-10* inhibitor restricted *CXCL8* expression in co-cultured cells (Red: macrophage; Green: GC cells). G. *IL-10* inhibitor limited the growth of subcutaneous tumors. H, I. *IL-10* inhibitor restricted the expression of CXCL8.

**Additional file 5.**

**Additional file 6.**

**Additional file 7.**

**Additional file 8.**

**Additional file 9.**

**Additional file 10.**

## Data Availability

The data are available from the sources listed in the manuscript—the TCGA and GEO data portal. The data used to support the findings of this study are available from the corresponding author upon request.

## Abbreviations

GC: Gastric cancer
TME: Tumor microenvironment
HIF-1α: Hypoxia-inducible factor-1α
TAMs: Tumor-associated macrophages
CXCL8/IL-8: C-X-C Motif Chemokine Ligand 8
IL-10: Interleukin 10
CXCR1/2: C-X-C Motif Chemokine Receptor 1/2
JAK: Janus Kinase 1
STAT1: Signal Transducer And Activator Of Transcription 1
GEO: Gene Expression Omnibus
TCGA: The Cancer Genome Atlas
PBMC: Peripheral blood mononuclear cell
FBS: Fetal bovine serum
IHC: Immunohistochemistry
qRT-PCR: Quantitative real-time polymerase chain reaction
ChIP: Chromatin immunoprecipitation
CCK8: Cell counting kit 8
OD: Optical density
DEGs: Differentially expressed genes
MACS: Magnetic-activated cell sorting
DFS: Disease-free survival
OS: Overall survival
