# Supplementary material for: A positive feedback loop between gastric cancer cells and tumor-associated macrophage induces malignancy progression
Source: J Exp Clin Cancer Res. 2022 May 14;41:174. doi: 10.1186/s13046-022-02366-6 (PMC9107227; doi:10.1186/s13046-022-02366-6)
Supplement: Supplementary file 1 — Additional file 1: Supplementary Table 1. Primer sequence. Supplementary Table 2. Antibodies information. [file 13046_2022_2366_MOESM1_ESM.docx]

Supplementary Table 1 Primer sequence

|  | Forward 5’-3’ | Reverse 5’-3’ |
| --- | --- | --- |
| CXCL8 | ATGACTTCCAAGCTGGCCGTGGCT | TCTCAGCCCTCTTCAAAAACTTCTC- |
| HIF-1α | GATCACCCTCTTCGTCGCTT | AAAGGCAAGTCCAGAGGTGG |
| IL-10 | CGCAGTGCAGAAGAGTCGAC | CCCGCTTGAGATCCTGAAATAA |
| STAT1 | GAGCACAGTGATGTTAGA | GCTGTTCTTGTTTCTGATC |
| β-actin | ATTGGCAATGAGCGGTT | CGTGGATGCCACAGGACT |
| Sh-STAT1#1 | CACCGCCTGACATTTACTTAGTACCCTCGAGGGTACTAAGTAAATGTCAGGC | AAAAGCCTGACATTTACTTAGTACCCTCGAGGGTACTAAGTAAATGTCAGGC |
| Sh-STAT1#2 | CACCGCAGAAGTCTCAAGCTATAAGCTCGAGCTTATAGCTTGAGACTTCTGC | AAAAGCAGAAGTCTCAAGCTATAAGCTCGAGCTTATAGCTTGAGACTTCTGC |

Supplementary Table 2 Antibodies information

|  | Source | Identifier |
| --- | --- | --- |
| CXCL8 | abcam | ab235584 |
| JAK | CST | #3332 |
| p-JAK | CST | #74129 |
| STAT1 | CST | #9172 |
| p-STAT1 | CST | #9177 |
| β-actin | CST | #9582S |
| HIF-1α | abcam | ab279654 |
| IL-10 | abcam | ab52909 |
| p65 | CST | #8242 |
| p50 | CST | #13586 |
| pho-p65 | CST | #3033 |
| Histone | abcam | #1791 |
| Second antibody | Santa Cruz | sc-2004 |
