# Supplementary figures and images for: A positive feedback loop between gastric cancer cells and tumor-associated macrophage induces malignancy progression
Source: J Exp Clin Cancer Res. 2022 May 14;41:174. doi: 10.1186/s13046-022-02366-6 (PMC9107227; doi:10.1186/s13046-022-02366-6)

A

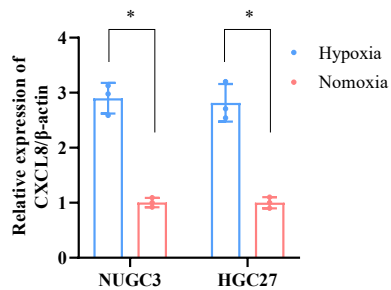

B

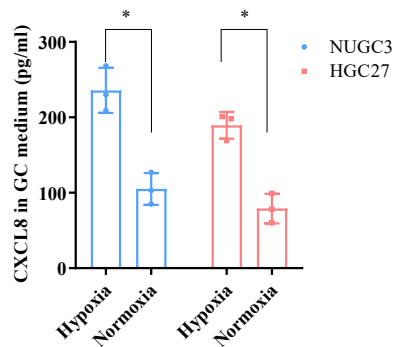

C

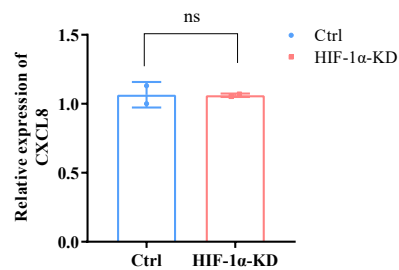

D

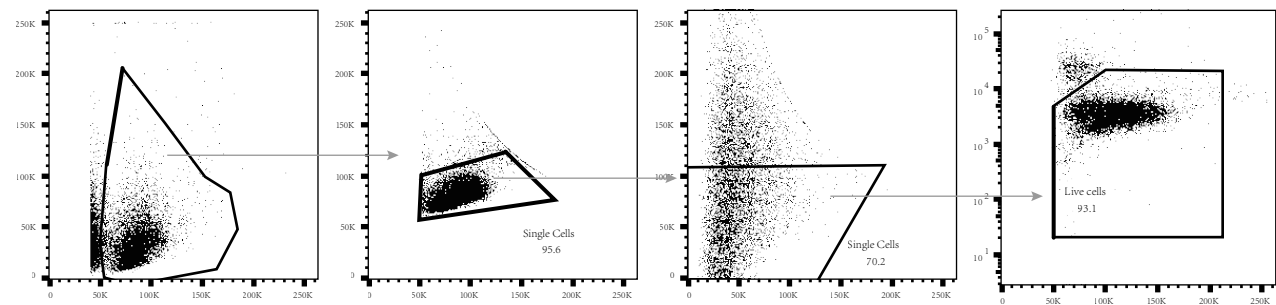

E

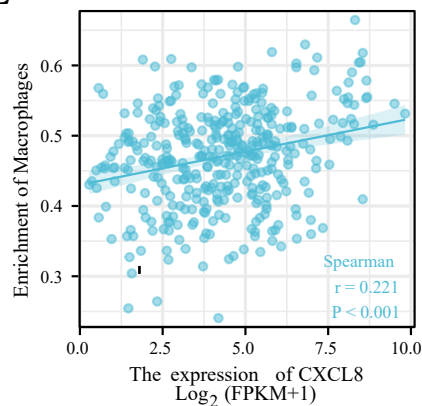

F

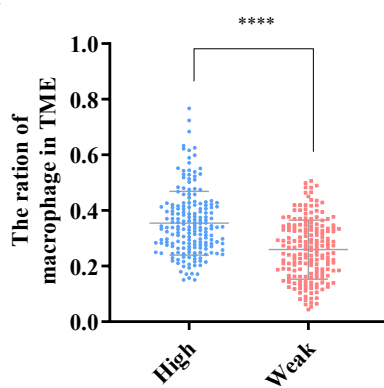

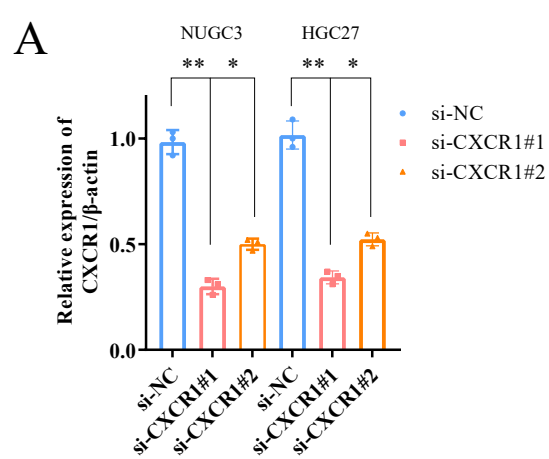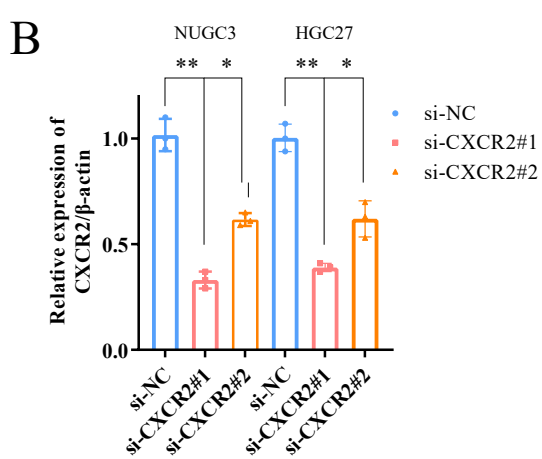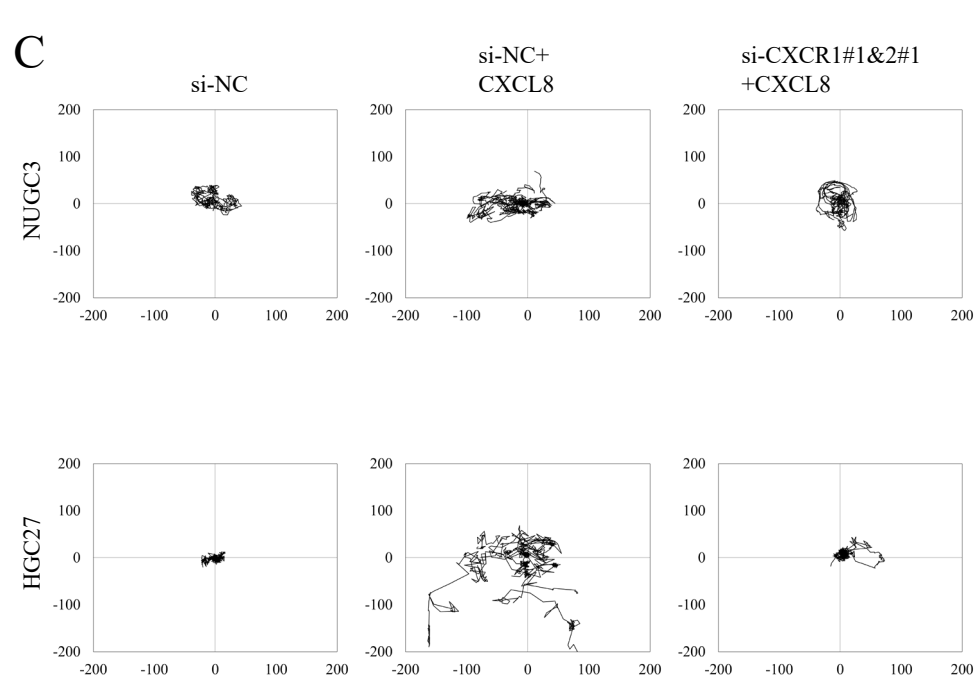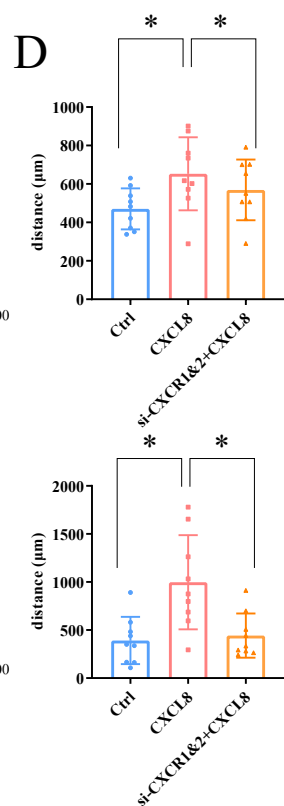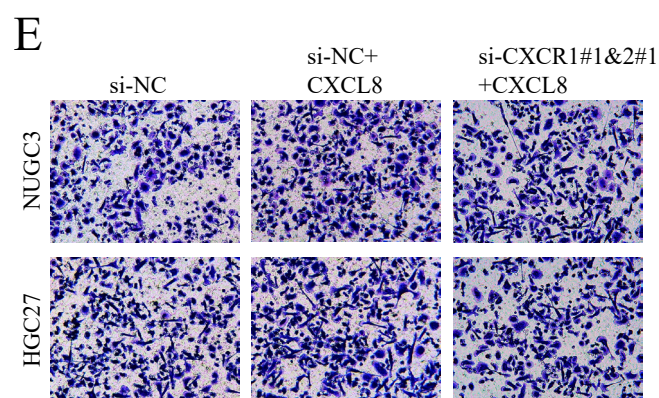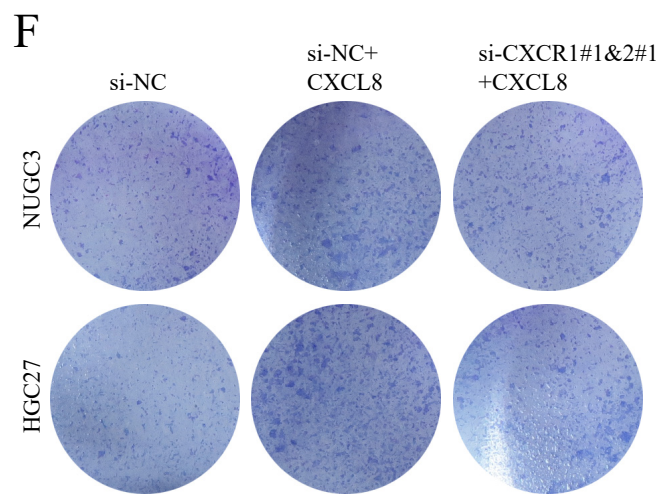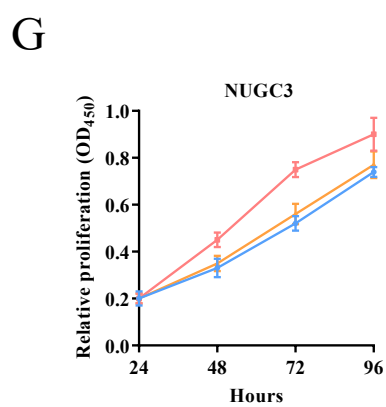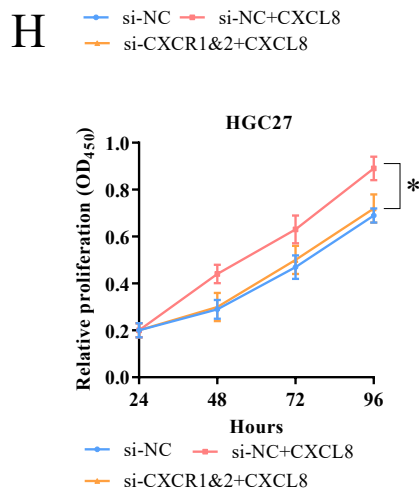

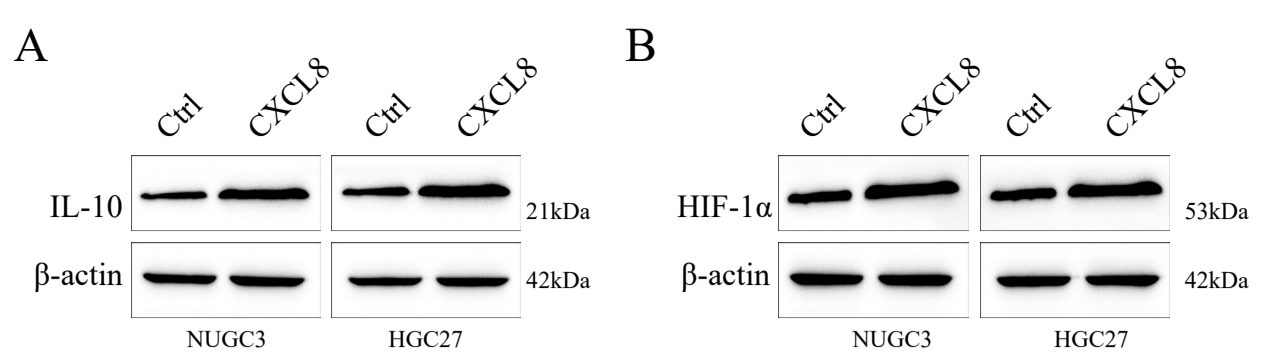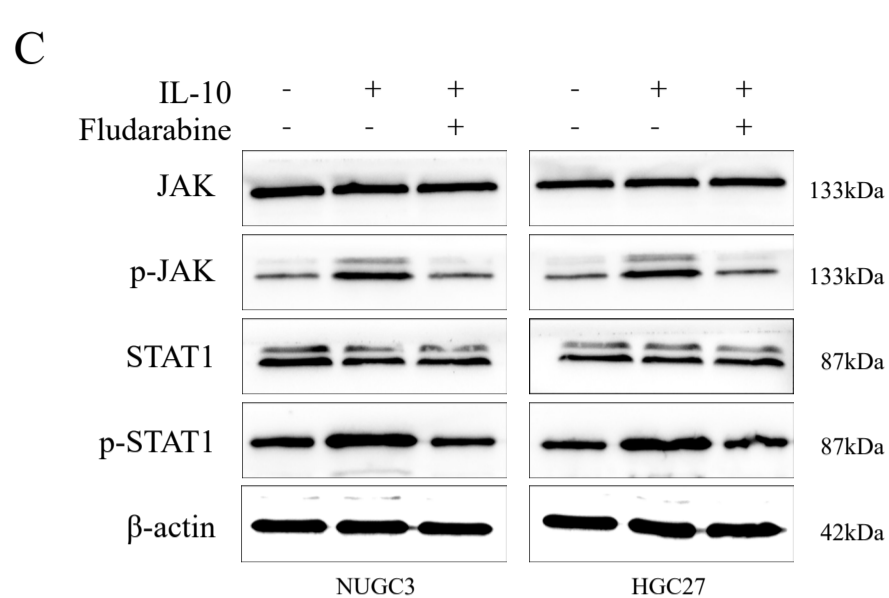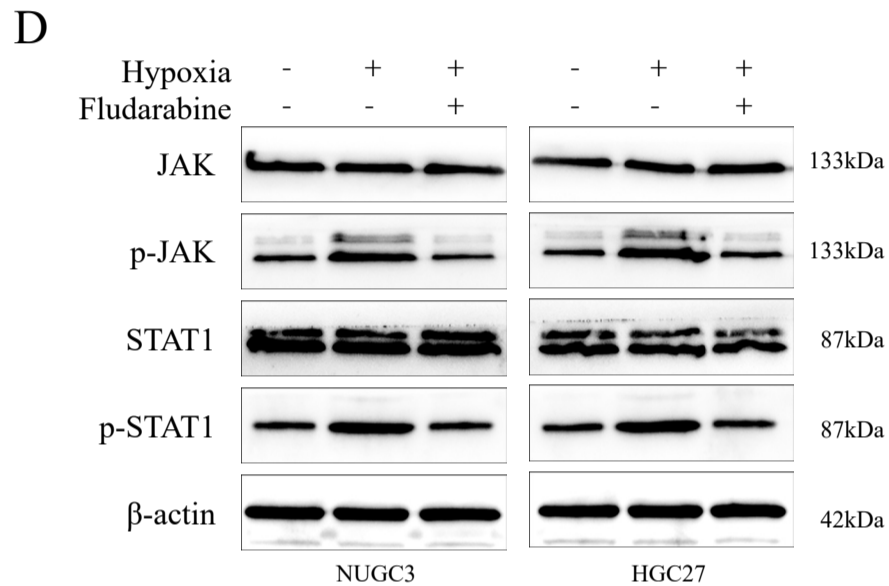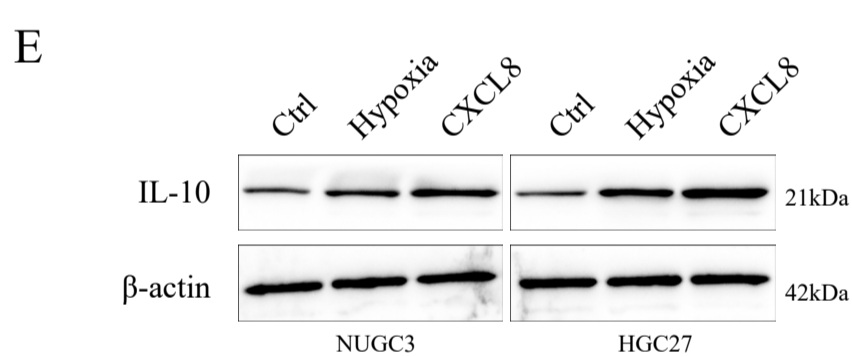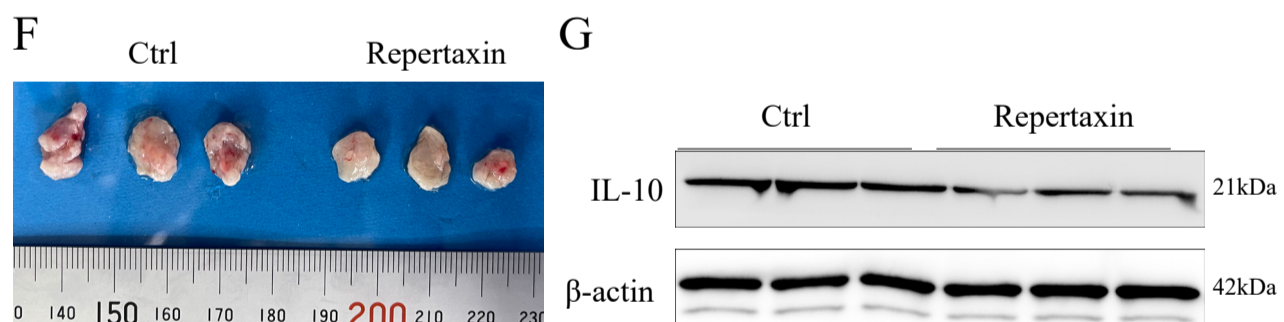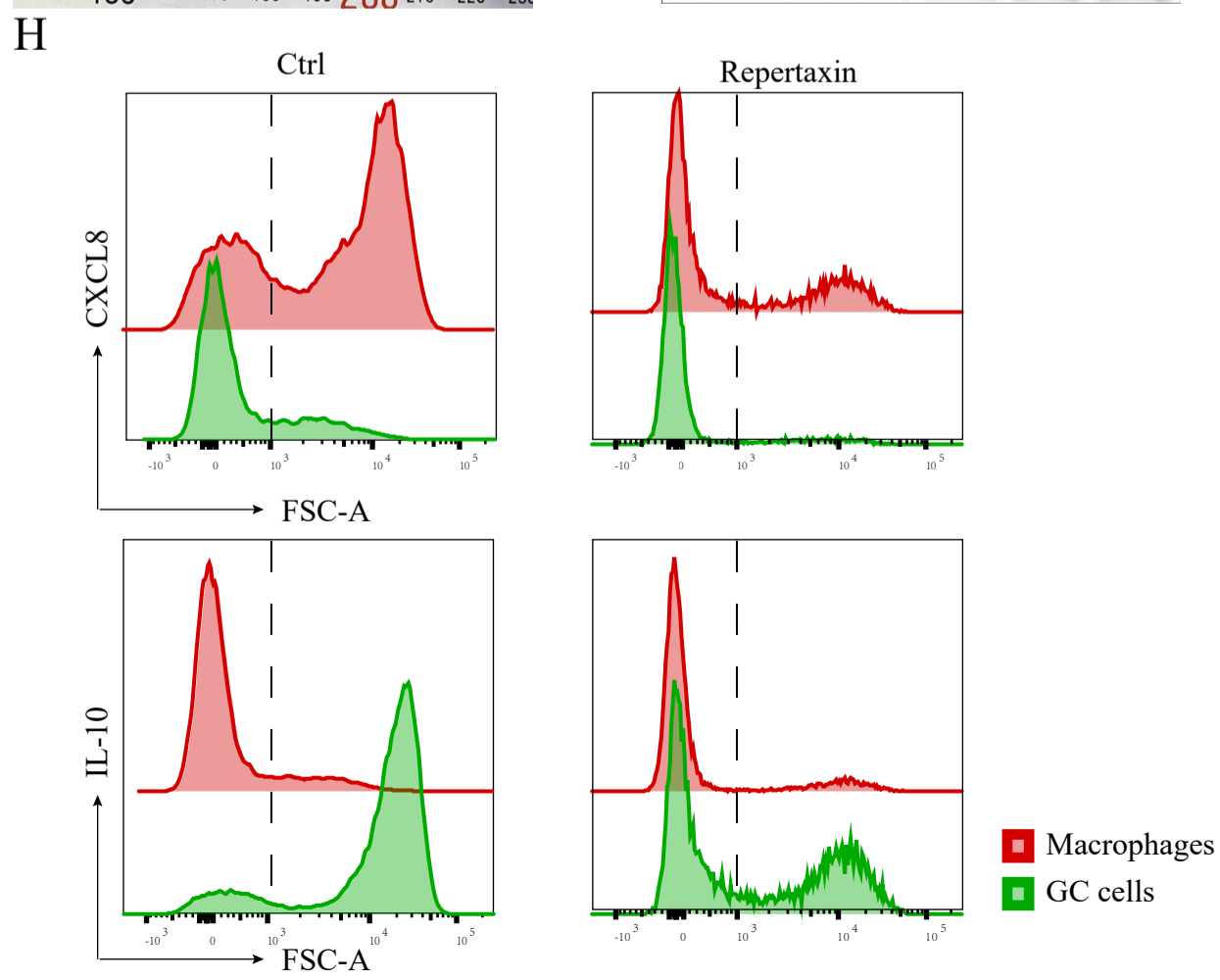

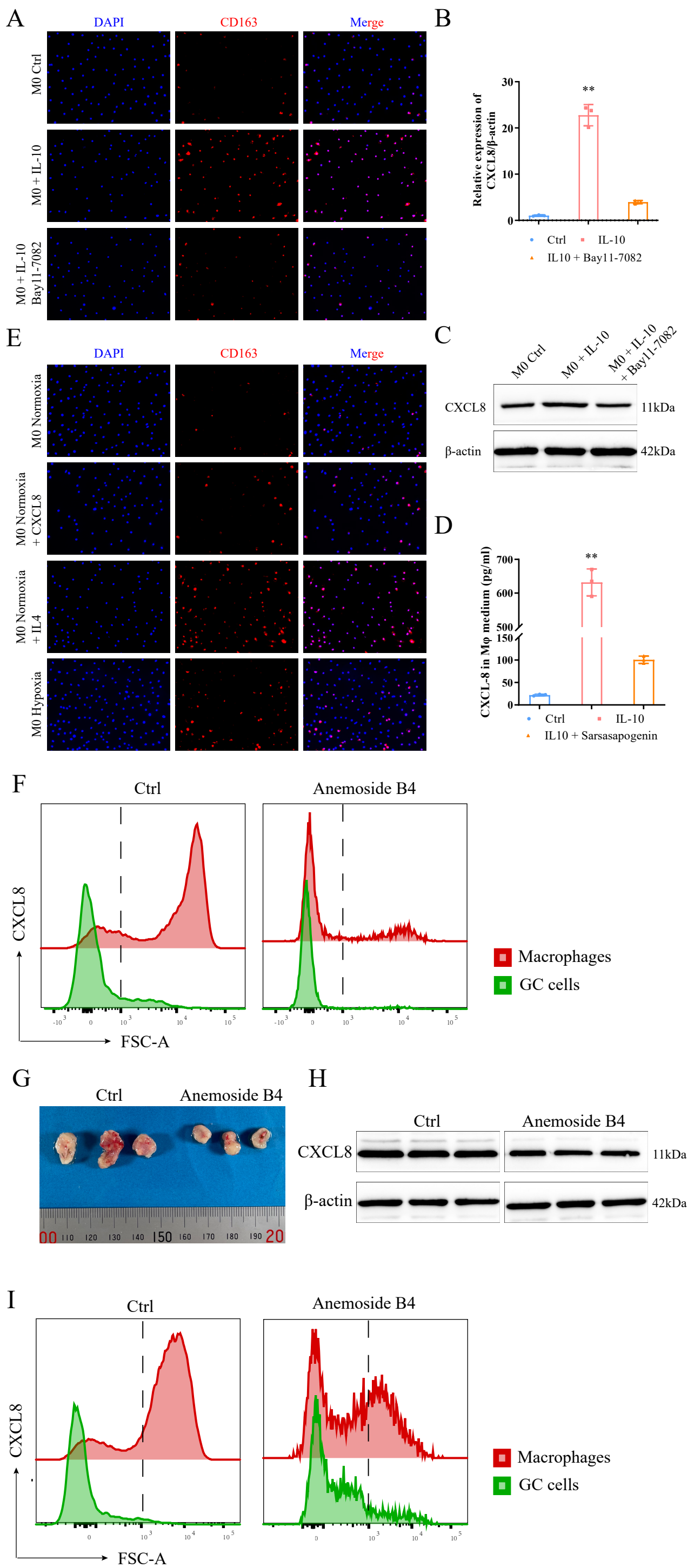

Supplement: Supplementary file 4 — Additional file 4: Supplementary Fig. 1. A. Hypoxia promoted CXCL8 expression in GC cells at mRNA level. B. Hypoxia induced the secretion of GC-derived cytokine CXCL8 secretion. C. The knockdown HIF-1α did not affect the expression of CXCL8. D. Cells were gated to exclude debris and dead cells. E. CXCL8 was positively correlated the proportion of infiltrating macrophages in TME. F. The infiltration ratio of macrophages in CXCL8-high group was significantly higher than that in CXCL8-weak group in TME. Supplementary Fig. 2. A, B. The siRNAs could effectively inhibit the expression of CXCR1 and CXCR2. C. Migration patterns of GC cells on Matrigel compared to cells cultured with CXCL8 alone and CXCL8 + si-CXCR1/2. D. Migration average distances of GC cells in each group. E. Transwell exhibited the differences in the number of permeable cells in each group. F, G, H. Colony formation assay and CCK-8 confirmed CXCL8 could promote GC proliferation which could be inhibited by si-CXCR1/2. Supplementary Fig. 3. A. CXCL8 promoted IL-10 expression in vitro. B. CXCL8 slightly promotes the expression of HIF-1α. C, D. IL-10 and hypoxia activated the JAK/STAT1 signal pathway. The STAT1 specific inhibitor (Fludarabine) could antagonize the effects. E. CXCL8 was more able to promote the expression of GC-derived IL-10 than hypoxia. F. CXCL8-CXCR1/2 inhibitor limited the growth of subcutaneous tumors. G, H. CXCL8-CXCR1/2 inhibitor restricted the expression of CXCL8 and IL-10 in tumor tissues. Supplementary Fig. 4. A. The NF-κB pathway inhibitor (Bay11–7082) could limit IL-10 induced M2 polarization. B, C. IL-10 upregulated CXCL8 expression, and it could be prevented by NF-κB pathway inhibitor (Bay11–7082). D. IL-10 could promote CXCL8 secretion, and Bay11–7082 could antagonize the effect of IL-10. E. CXCL8 and hypoxia could slightly induce macrophage M2 polarization. F. IL-10 inhibitor restricted CXCL8 expression in co-cultured cells (Red: macrophage; Green: GC cells). G. IL-10 inhibito [file 13046_2022_2366_MOESM4_ESM.pdf]
